# The Impact of Inventory Management on Stock-Outs of Essential Drugs in Sub-Saharan Africa: Secondary Analysis of a Field Experiment in Zambia

**DOI:** 10.1371/journal.pone.0156026

**Published:** 2016-05-26

**Authors:** Ngai-Hang Z. Leung, Ana Chen, Prashant Yadav, Jérémie Gallien

**Affiliations:** 1 Tepper School of Business, Carnegie Mellon University, Pittsburgh, Pennsylvania, United States of America; 2 Analytics Operations Engineering, Boston, Massachusetts, United States of America; 3 William Davidson Institute, Ross School of Business and School of Public Health, University of Michigan, Ann Arbor, Michigan, United States of America; 4 London Business School, Regent’s Park, London, NW1 4SA, United Kingdom; Tulane University School of Public Health and Tropical Medicine, UNITED STATES

## Abstract

**Objective:**

To characterize the impact of widespread inventory management policies on stock-outs of essential drugs in Zambia’s health clinics and develop related recommendations.

**Methods:**

Daily clinic storeroom stock levels of artemether-lumefantrine (AL) products in 2009–2010 were captured in 145 facilities through photography and manual transcription of paper forms, then used to determine historical stock-out levels and estimate demand patterns. Delivery lead-times and estimates of monthly facility accessibility were obtained through worker surveys. A simulation model was constructed and validated for predictive accuracy against historical stock-outs, then used to evaluate various changes potentially affecting product availability.

**Findings:**

While almost no stock-outs of AL products were observed during Q4 2009 consistent with primary analysis, up to 30% of surveyed facilities stocked out of some AL product during Q1 2010 despite ample inventory being simultaneously available at the national warehouse. Simulation experiments closely reproduced these results and linked them to the use of average past monthly issues and failure to capture lead-time variability in current inventory control policies. Several inventory policy enhancements currently recommended by USAID | DELIVER were found to have limited impact on product availability.

**Conclusions:**

Inventory control policies widely recommended and used for distributing medicines in sub-Saharan Africa directly account for a substantial fraction of stock-outs observed in common situations involving demand seasonality and facility access interruptions. Developing central capabilities in peripheral demand forecasting and inventory control is critical. More rigorous independent peer-reviewed research on pharmaceutical supply chain management in low-income countries is needed.

## Introduction

### Background

Stock-outs of essential medicines at the clinic level are an important and widely acknowledged public health problem in sub-Saharan Africa (SSA) with a recognized negative impact on morbidity, mortality and disease epidemiology [1,2]. Many possible causes have been cited, including procurement financing and processes, supply capacity, communication and road infrastructure, distribution resources and planning methods, personnel staffing and training, coordination among stakeholders [3–5]. Reported related interventions include technical or managerial training [6], visibility of stock levels with SMS messaging [7], re-organization of distribution activities [8], supply-chain structure [9], and others. This study seeks to characterize the specific impact of inventory management policies currently used in many low-income countries, leveraging the recent intervention in Zambia's public pharmaceutical supply chain described by Vledder et al. and further discussed below (the *2009/10 pilot*) [9].

### Zambia's public pharmaceutical supply chain and the max-min inventory policy

**Fig 1:**
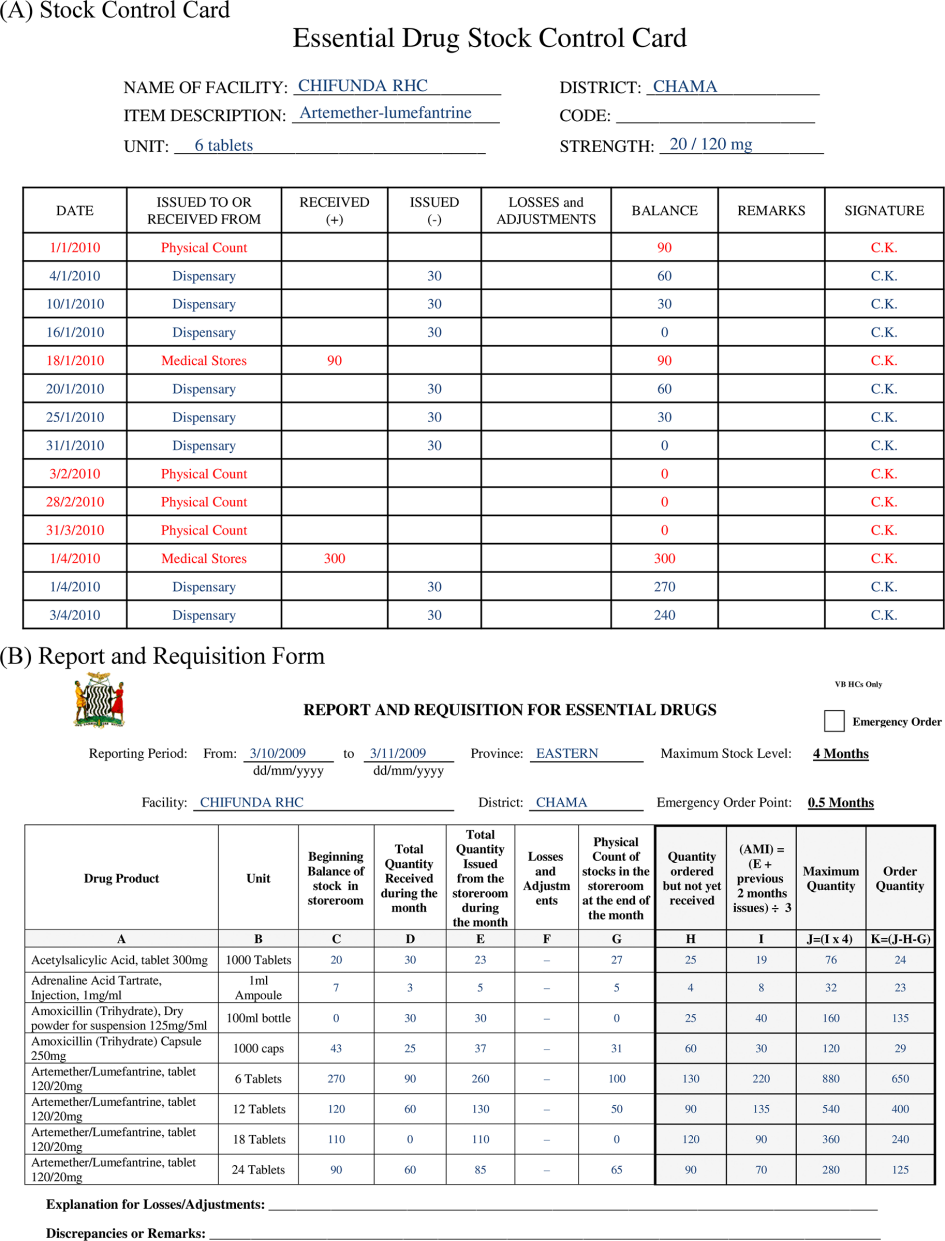
Main paper forms supporting inventory control system in Zambia. (A) Stock Control Card completed for every product in each storage facility. (B) Request and Requisition Form transmitted upstream to calculate and communicate monthly replenishment orders. Populating data shown for illustration purposes only. Only some of the pre-printed product descriptions are shown.

Most patients in Zambia receive medicines freely through the public health system. It involves procurement of medicines with financing from the Government of Zambia and external donor support, primary monthly distribution from a national warehouse in Lusaka to approximately 70 district stores and 20 hospitals, and secondary monthly distribution from district stores to approximately 1500 health clinics [10]. Availability of medicines in clinics is relatively low and typical of SSA [11]. Stock accounting is performed by clinic staff who record inventory transactions and historical storeroom stock levels for every product on a form often called stock card (Fig 1A). Inventory management follows guidelines currently recommended by the USAID|DELIVER project, which are also applied in many low-income countries [12]. They involve a periodic review base-stock policy [13] known as max-min inventory control, and the monthly completion and upstream communication by every facility of request and requisition forms (Fig 1B). Monthly replenishment quantities are determined as the difference between a target (maximum stock level) and the sum of current on-hand and pipeline inventory. That replenishment target is calculated as a multiple of average monthly issues over the previous two to four months [12]. For products with demand seasonality such as anti-malarials, USAID|DELIVER recently recommended other methods for calculating replenishment targets [14,15].

### The 2009/2010 supply chain pilot

The government of Zambia led from April 2009 to April 2010 a quasi-randomized field experiment in 16 districts to evaluate two supply chain models (the *2009/10 pilot*). Intervention A maintained intermediary stock with picking and packing activities in district stores; Intervention B converted district stores into cross-docking points for shipments pre-packaged for the clinics at the national warehouse. For intervention A, maximum stock levels were set to two months in clinics and three months in district stores. For intervention B, maximum stock levels in clinics were set to four months. In 12 intervention districts a new employee (commodity planner) was solely dedicated to inventory management. In the national warehouse, a team was dedicated to reviewing submitted replenishment requests. This staff and process ensured a high adherence to the prescribed inventory control policies. Specific procurement arrangements ensured high levels of stock availability at the central warehouse during the pilot period for a sample set of products selected for evaluation. Primary analysis discussed in Vledder et al. focused on impact evaluation over Q4 2009. It reports a substantial reduction of the average number of days without stock for these products in intervention B compared with intervention A and control facilities (Table a in S1 File provides results for artemether-lumefantrine products). On this basis the government of Zambia decided later in 2010 to progressively deploy district cross-docking to the entire country [9].

## Methods

### Data collection

The four dosage forms (6, 12, 18 and 24 tablet strips) of artemether-lumefantrine (AL) were selected because they involve representative distribution challenges (seasonality, distribution to all clinics) and are important to public health. 975 photographs of their stock cards in 145 clinics were taken by commodity planners during regular district tours between June 2009 and June 2010. Database transcription was performed by a data entry firm using a double-key process. Probability of stock-out was estimated for each product and day in this dataset as the fraction of clinics with no inventory to the total number of clinics with available records.

All 17 clinics with time coverage of at least nine months in this dataset were selected from five districts for demand estimation purposes. In each of these clinics, raw demand rate on any day with at least one box in recorded inventory was calculated for every product. That quantity was computed as the number of tablets in a box divided by the number of days between the last and next issues of a box. To smooth discontinuities and estimate censored demand, a triple centered moving average operator with successive half-widths 40, 30 and 20 days of non-censored data was applied [16]. Because of common demand substitution between AL products (which contain identical pills), their individual demand estimates were summed across products and these sums were then averaged across clinics. This resulted in an estimate of average daily demand for all AL products in a typical clinic. This total demand was split into demand estimates for each product using proportions equal to the fractions of issues observed in the stock card dataset.

To estimate demand distributions, several parametric probability distributions including normal, Poisson, geometric, lognormal and negative binomial were fitted through maximum likelihood to historical demand from the stock card dataset. Considering final likelihood and parameter values of fitted distributions led to the selection of the lognormal distribution with a coefficient of variation of 50% to represent weekly demand (see Section D in S1 File for more details).

Distribution from the national warehouse to clinics consist of a primary stage (national warehouse to districts) and a secondary stage (districts to clinics). Primary distribution follows a fixed and reliable monthly schedule, with a lead-time of approximately 2 weeks. Records of secondary lead-times to 212 clinics were obtained from monthly reports submitted by commodity planners between May 2009 and June 2010. Staff in 212 clinics was surveyed for subjective estimates of the probability that in any week of each month a shipment ready for their facility at the district could be delayed because of weather-related access problems. Secondary lead-times without access problems were modeled as a geometric random variable with first moment set to the sample mean estimated from historical data. Seasonal access problems were captured by a Bernoulli random variable with time-dependent success probability given by the surveyed subjective probability estimates.

Monthly closing stock levels of all AL products in the national warehouse between November 2007 and February 2011 were obtained from its stock management software. Between June 2009 and May 2010 the stock of all AL products available there for distribution was always higher than 800,000 adult regimens equivalent, where 100 tablet strips of AL 6 (respectively AL 12, AL 18 and AL 24) = 25 (respectively 50, 75 and 100) adult regimens equivalent.

### Simulation model

Our study relies on a discrete-event simulation model of the inventory of AL products in a typical clinic operated under max-min inventory control. This model was developed in the Java programming language [17]. This methodology involves the computer representation of a dynamic random system as a discrete sequence of events in time, and is common in research focusing on inventory management [18]. In our case the main simulated variables of interest are the inventory level of AL products in a typical clinic and the outstanding replenishment orders sent by that clinic to an upstream facility (district or central warehouse). The relevant events include replenishment orders, demand for products by patients, and deliveries. The dynamics captured by the simulation model specify when these events occur on a simulated timeline of weekly time increments, reflecting for example a random lead-time between order and delivery. They also capture what happens to the inventory and outstanding replenishment order variables when each of these events occurs, reflecting the specific calculation of replenishment quantities as part of the max-min inventory control policy, or the random realizations of demand. Consistent with relevant national warehouse stock records, our simulation model assumes an infinite amount of inventory available upstream. Its initial simulation state has no available inventory and no outstanding replenishment order. Subsequent simulation time steps are one week and involve the following sequence:

1. Any replenishment order scheduled to be delivered that week is credited to the inventory level;
2. If that week is the first of a calendar month (assumed monthly schedule for primary distribution), an order quantity is calculated according to the min-max policy being simulated. The future delivery week of that order is generated by adding a realization of the random delivery lead-time described above to an assumed order transmission delay of two weeks;
3. Demand is generated as the realization of a lognormal random variable with time-dependent mean estimated as discussed above and coefficient of variation equal to 50%. Stock issues are calculated as the minimum of inventory level and demand, unmet demand as the difference between demand and issues when positive, and the inventory level for the next simulated week is calculated by subtracting issues.

Each simulation replication in our study (that is each simulated history or path realization of the inventory and order variables over time) involves a warm-up period of two years and a data collection period of three years after that. This warm-up period is designed to eliminate the impact of the arbitrary initial simulation state on the results. For each scenario considered, 100,000 simulation replications form the basis of reported statistical results. The simulated performance measures include: service level, calculated as the fraction of demand satisfied from available inventory over the total demand occurring during the data collection period; and average and maximum inventory level over the data collection period. Simulated inventory management policies are all variants of the max-min inventory policy using different methods for calculating the replenishment target. These methods are characterized by whether the target is calculated based on past average monthly issues (I) or average monthly demand (D); the historical interval of time over which these averages are calculated (defined in relation to the current month); and the multiple of these average monthly estimates used to calculate the replenishment target. Our notation follows, for example 4 x I[-3,0] denotes a replenishment target equal to four times average monthly issues calculated over the previous three months, while 5 x D[-12,-9] denotes five times average monthly demand calculated over the next three calendar months from the year before. We simulated the baseline policy 4 x I[-3,0] used during the 2009/10 pilot and evaluated the impact of its maximum stock multiple by also considering 3 x I[-3,0]; 5 x I[-3,0]; and 6 x I[-3,0]. We also considered the following policy modifications recommended for malaria supply chains by the USAID|DELIVER project [14]: 4 x I[-1,0]; 4 x I[-6,0]; 4 x I[-12,0]; 4 x I[-12,-9]. Finally, we assessed the specific impact of past issues as opposed to demand information by simulating the policies 4 x D[-1,0]; 4 x D[-3,0]; 4 x D[-6,0]; 4 x D[-12,0]; and 4 x D[-12,-9]. We were not able to simulate the inventory replenishment policy based on seasonality indices that is described in the recent concept paper by Watson et al., because its specifications assume perfect advance knowledge of delivery lead-times and seasonal access restrictions [15].

Sensitivity analysis was performed with respect to demand seasonality by considering mean demand curves obtained by vertically re-scaling the estimated baseline while keeping annual average constant. We considered values from 1 to 3·4 for the resulting seasonality factor (ratio of peak demand mean to average mean through the year). Sensitivity with respect to delivery lead-times was also performed by considering values for the mean secondary lead-time distribution ranging from 2·2 to 5·4 weeks.

### Model validation

An important component of our simulation-based methodology is the validation of our model out-of-sample, which was performed to evaluate its realism and predictive accuracy. Specifically, actual field measurements of stock-outs were compared with their simulated values, which were obtained independently under model settings reproducing the conditions believed to characterize the field data generation process. This required a modified experimental design considering the four AL products separately because it was not clear how to meaningfully aggregate their historical stock-out records. Specific initial conditions were also used because the pilot set-up phase involved a large upfront delivery of inventory to participating health clinics before the implementation of policy 4 x I[-3,0]. Analysis of stock card data reveals a variation of this initial delivery date across clinics between May 1 and September 30 2009. The quantity received then also varied across products and was estimated at 0·8, 1·8, 1·5 and 0·95 times the target stock level for product AL 6, 12, 18 and 24, respectively. To capture these observations, validation experiments involved for each replication a starting date of May 1 2008 with initial inventory equal to eight weeks of future demand, a simulation of the policy 2 x I[-3,0] until a date uniformly distributed between May 1 and September 30 2009, an initial pilot set-up order on that date for a quantity equal to 0·8, 1·8, 1·5 or 0·95 times the target stock level corresponding to the product considered, and a subsequent simulation of monthly orders calculated according to the baseline pilot policy 4 x I[-3,0] until May 1 2010.

## Results

### Parameter estimation

**Fig 2:**
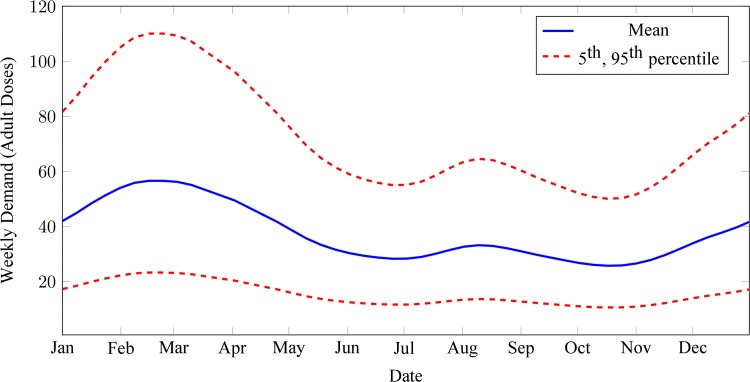
Estimated mean, 5^th^ and 95^th^ percentiles of combined weekly demand for all artemether-lumefantrine (AL) products at an average health clinic. Statistics computed from stock card data of 17 clinics with at least nine months of consecutive demand data in stock card dataset, assuming no annual trend.

Key statistics of the estimated demand model for all AL products include an annual average of 37 adult-equivalent regimens per week, with maximum and minimum averages of 57 and 26 adult-equivalent regimens per week in late February and late October, respectively (Fig 2). Estimated proportions of demand for AL 6, 12, 18 and 24 were 0·48, 0·27, 0·30 and 0·52, respectively.

**Fig 3:**
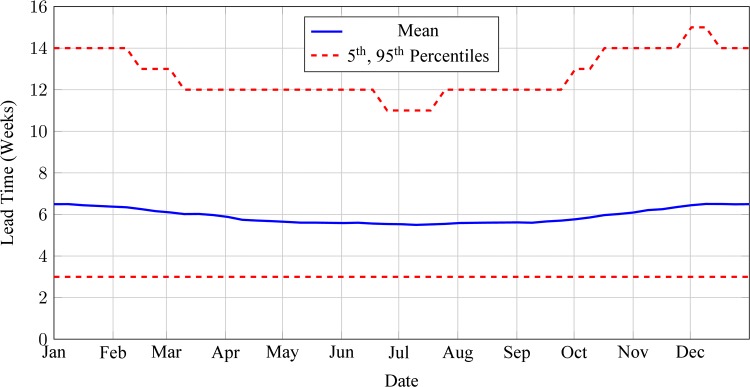
Estimated mean, 5^th^ and 95^th^ percentiles of delivery lead-times between the central warehouse and an average health clinic. Estimates based on record of lead-times to 212 clinics between May 2009 and June 2010 and surveys of subjective probabilities of shipment delays due to weather-related access problems, assuming no annual trend.

Estimated mean secondary lead-time in periods without access interruptions was 4 weeks. Average subjective probabilities provided for clinic accessibility were 0·78, 0·76, 0·78, 0·85, 0·93, 0·97, 0·97, 0·98, 0·99, 0·99, 0·95 and 0·85 for the months ranging from January to December, respectively. Key statistics of the resulting distribution lead-time model include an annual average of 4 weeks, with maximum and minimum averages of 4·9 and 3·9 weeks in December and July, respectively (Fig 3).

### Field stock-outs and model validation

**Fig 4:**
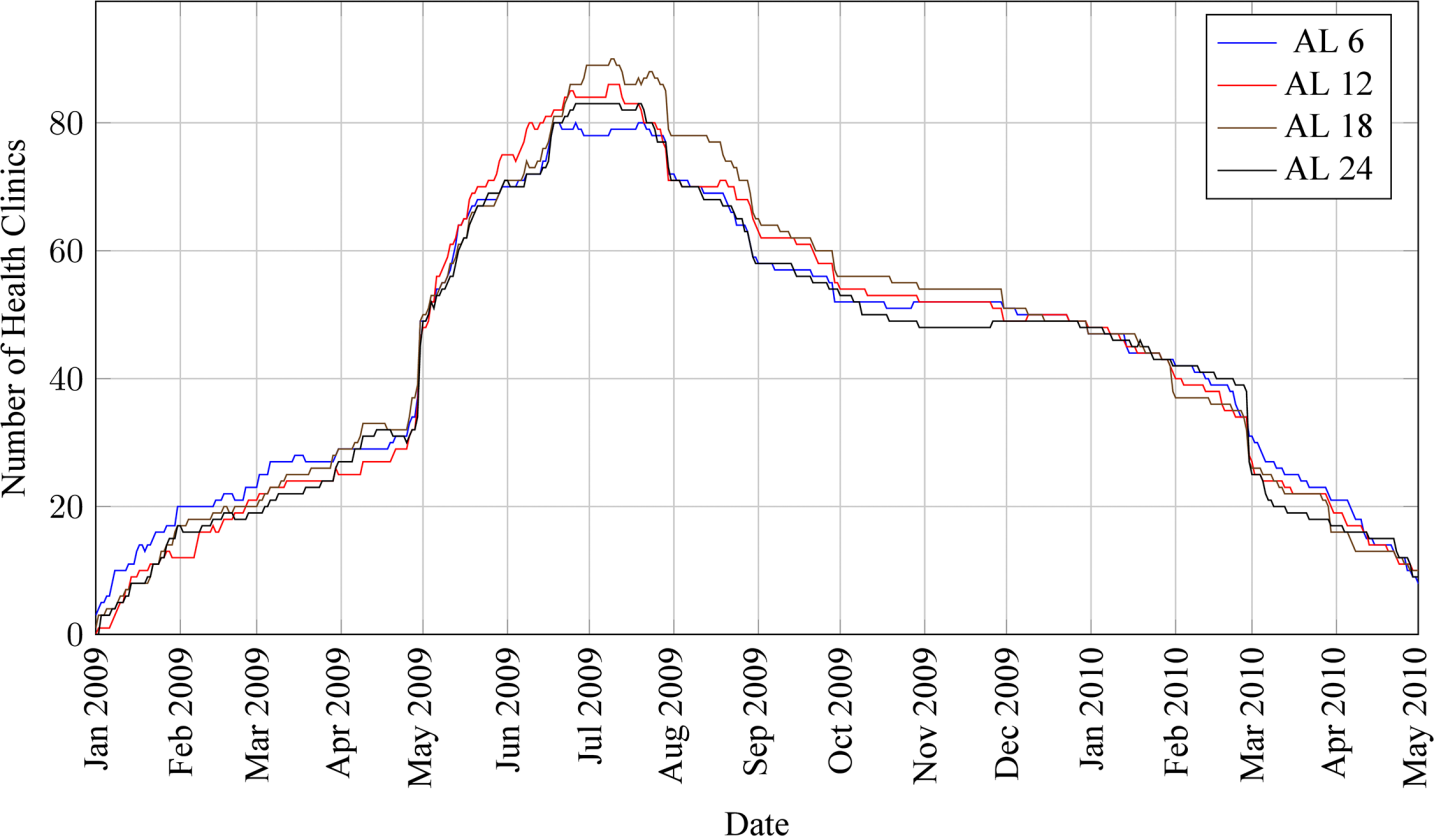
Number of health clinics for which artemether-lumefantrine (AL) products stock data was obtained from stock card collection process, January 2009 –May 2010.

The number of clinics for which historical stock data was obtained was similar across AL products, ranging from around 20 in March 2009 to around 80 in July 2009 (Fig 4). The variability of this clinic sample size over time results from a number of factors that were difficult to control during the data collection campaign. These include field availability and functionality of digital cameras (battery and memory card issues); schedule of clinic visits by commodity planners; understanding of, and adherence to, the data collection protocol; proper maintenance of stock records by clinics over time; timeline of participation by commodity planners; etc.

**Fig 5:**
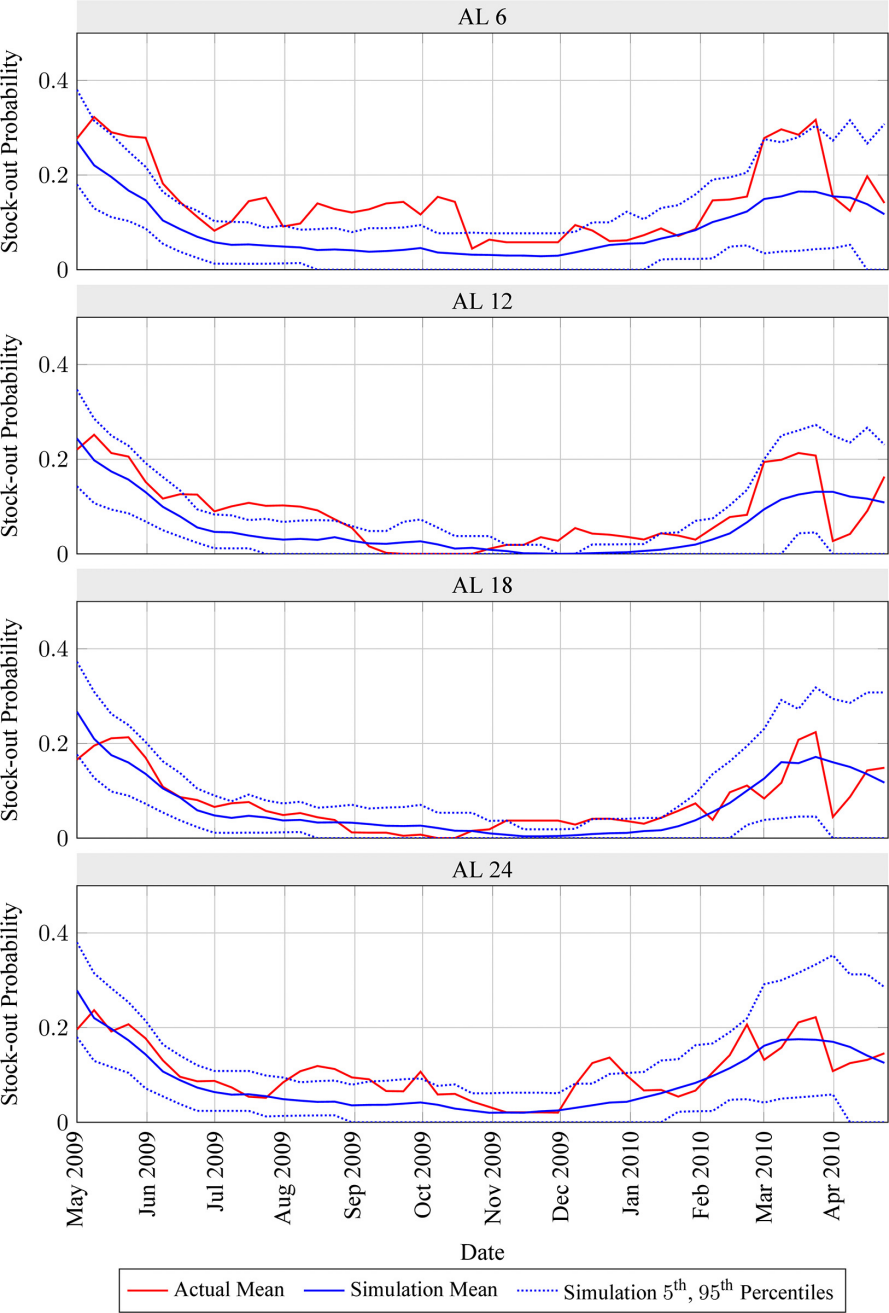
Actual and simulated stock-out probabilities for artemether-lumefantrine (AL) products, observed through field stock cards (mean), and as predicted using simulation model (mean, 5th and 95th percentiles). Actual stock-out probabilities estimated as the fraction of surveyed health facilities without stock. Mean simulated stock-out probabilities estimated as the fraction of 100,000 replications without stock. Plotted 5th and 95th percentiles correspond to a binomial distribution with success probability equal to the mean simulated stock-out probability and number of trials equal to the clinic sample size shown in Fig 4.

The proportion of stocked-out clinics calculated from historical stock data consistently decreased over the first two quarters of the 2009/10 pilot, from around 20% (30% for AL 6) in May 2009 to 5% or less in October-November 2009 (Fig 5). After approximately two months at this low level, this proportion increased again starting from January 2010 (December 2009 for AL 24) however, peaking at approximately 20% (30% for AL 6) sometime in late February or March 2010. Data available for April and May 2010 suggests that the fraction of stocked-out clinics began to decrease again over this period.

Actual stock-out proportions for all AL products mostly lie between the 5^th^ and 95^th^ percentiles of simulated stock-out proportions, with excesses above the 95^th^ percentile (most notably for AL 6 between July and October 2009) being of relatively small amplitude (Fig 5). Another positive model validation follows from comparing the simulated number of days without stock during Q4 2009 with the corresponding actual values estimated from the field measurements organized independently for the evaluation of the 2009/10 pilot (see Section B in S1 File for details).

### Simulation experiments

**Fig 6:**
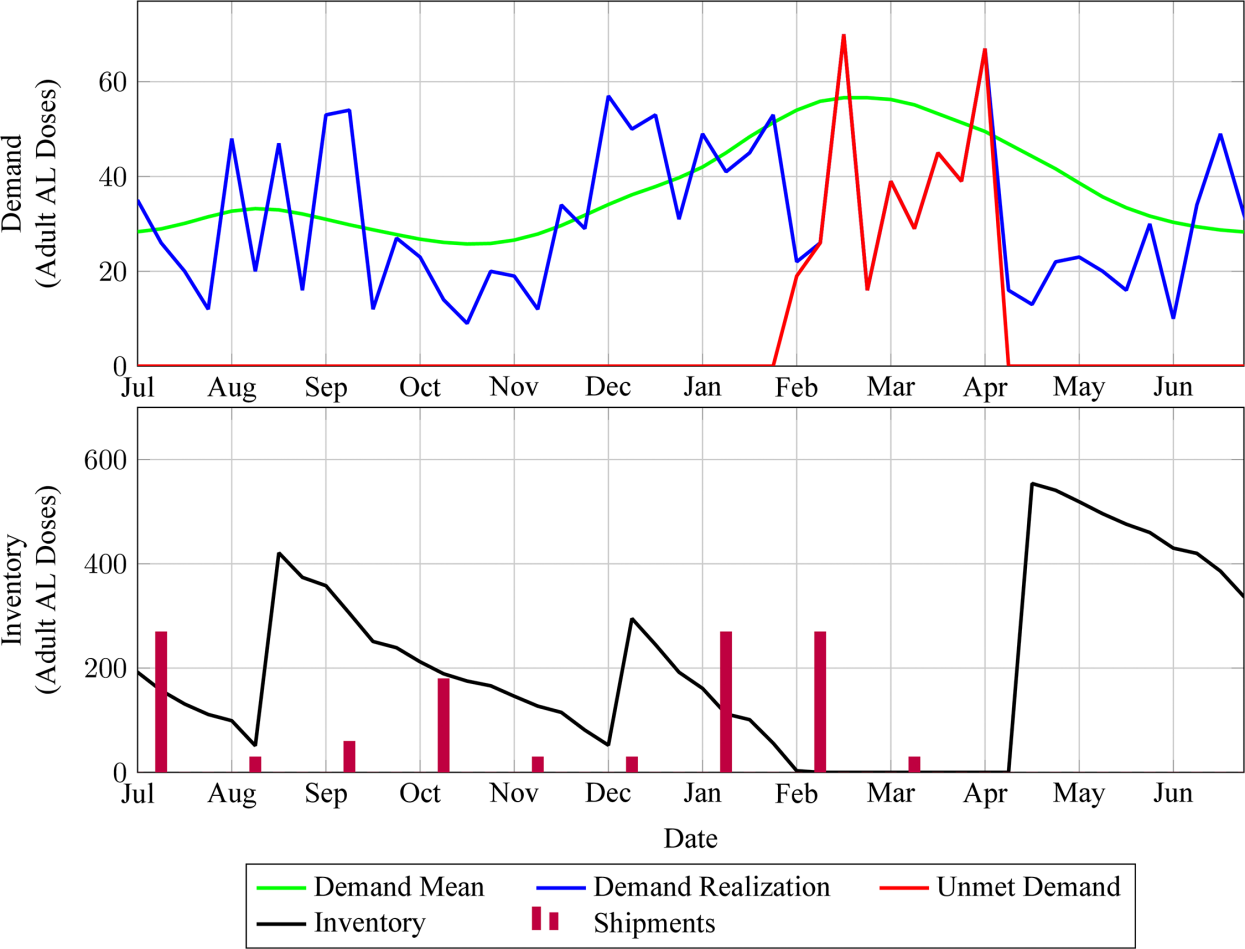
Randomly selected simulation sample path of historical max-min inventory control policy. Simulated data for policy 4 x I[-3,0] shown after warm-up period of one hundred years chosen to eliminate impact of initial conditions. Demand mean data from Fig 2.

Randomly selected simulation sample paths under policy 4 x I[-3,0] (Fig 6) explain the stock-outs increase in Q1 and Q2 2010 seen in Fig 5: as demand starts to increase in December (beginning of malaria season), the estimation of monthly issues then is based on the low historical demand of the three previous months so that the December shipment is small. While the January and February shipments are substantially larger (because inventory has been depleted at a faster rate during December), these are only received in April because of longer delivery lead-times during the rainy season. Consequently the facility stocks out from February to early April (peak demand period), but receives enough inventory to cover approximately five months of demand in May, when the malaria season finishes and this quantity of medicines is no longer needed.

**Fig 7:**
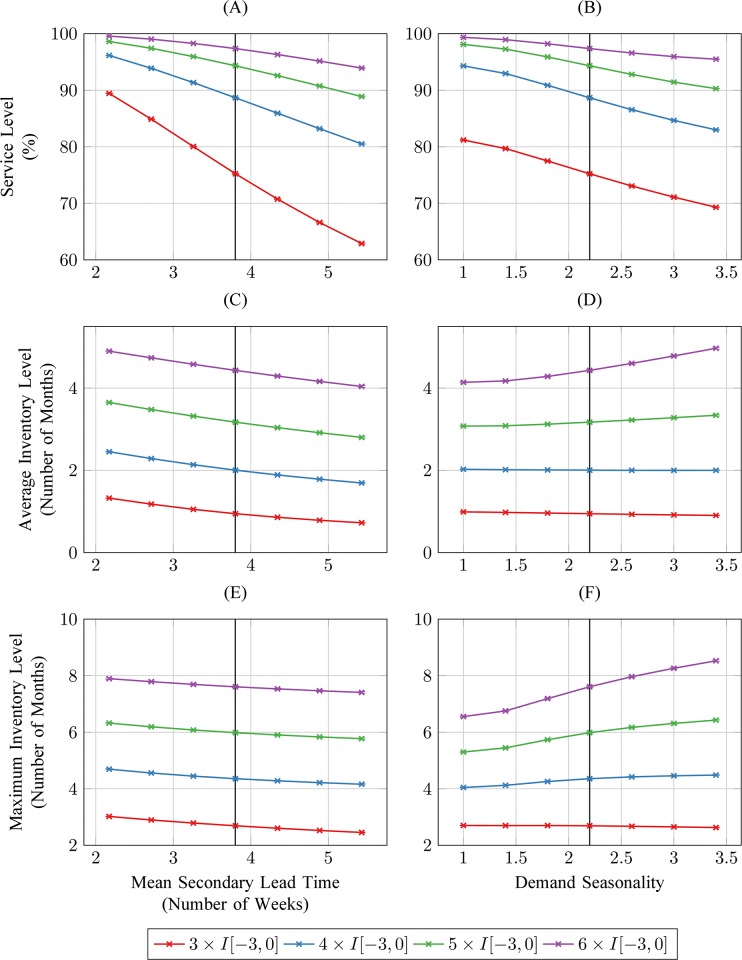
Simulated average service level, average and maximum inventory levels when monthly replenishment targets are calculated as different multiples of average monthly consumption. Each simulation replication includes a warm-up period of two years and a data collection period of three years. Vertical whiskers around each data point show 95% confidence intervals around mean simulation estimates, but are barely visible because they are calculated from 100,000 simulation replications. Estimated baseline values are indicated with a vertical line.

Annual service level average of historical policy 4 x I[-3,0] over multiple replications and under environmental conditions estimated from data is approximately 88% (Fig 7A and 7B), with average and maximum yearly inventory levels of 2 and 4·3 months of demand, respectively (Fig 7C–7F). Sensitivity analysis on the multiple of average past monthly issues used in the replenishment target calculation shows a strong impact on both service level and inventory levels. Specifically, increasing the target multiple from 4 months to 5 and 6 months increases service levels from 88% to 95% and 97% (Fig 7A and 7B) and maximum inventory levels from 4·3 months to 6 and 7·8 months, respectively (Fig 7E and 7F). In addition, decreasing the target multiple from 4 to 3 months reduces the average service level by more than 12 percentage points (Fig 7A and 7B), and decreases average and maximum inventory levels by approximately 50% (Fig 7C–7F). Increasing the delivery lead-time decreases both service level and inventory levels, and increasing the demand seasonality factor decreases service level but tends to increase inventory levels. In particular, an increase of the average delivery lead-time by only one week reduces the service level of the historical policy 4 x I[-3,0] down to 83% (Fig 7A).

**Fig 8:**
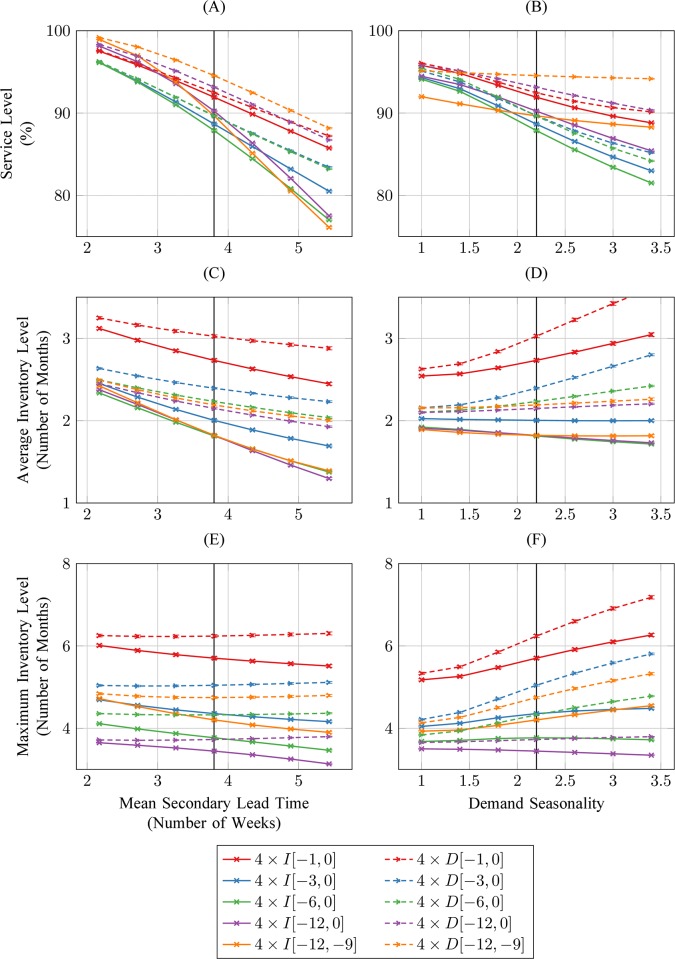
Simulated average service level, average and maximum inventory levels when monthly replenishment targets are calculated with different methods for estimating historical average monthly consumption and demand. Each simulation replication includes a warm-up period of two years and a data collection period of three years. Vertical whiskers around each data point show 95% confidence intervals around mean simulation estimates, but are barely visible because they are calculated from 100,000 simulation replications. Estimated baseline values are indicated with a vertical line.

The changes in the calculation method for average monthly issues recommended by USAID | DELIVER [14] have a limited impact on service level (reduction from 88% to 87·5% for 4 x I[-6,0], increases to 89%, 90% and 92% for 4 x I[-12,-9], 4 x I[-12,0] and 4 x I[-1,0], respectively–Fig 8A and 8B). All these recommended changes except 4 x I[-1,0] result in minor decreases of average inventory, however policy 4 x I[-1,0] increases average inventory from 2 to 2·7 months (Fig 8C and 8D) and maximum inventory from 4·3 to 5·7 months (Fig 8E and 8F). Using past monthly demand in replenishment target calculations increases service level by less than 2% for 4 x D[-1,0], 4 x D[-3,0] and 4 x D[-6,0], by 2·5% for 4 x D[-12,0] and by 5% for 4 x D[-12,-9] (Fig 8A and 8B). The service level of all considered policies degrades as lead-time increases, and in particular the best-performing policy 4 x D[-12,9] sees unmet demand increase from 5% to 10% when average lead-time increases by only one week (Fig 8A). All policies except 4 x D[-12,9] and 4 x I[-12,0] see their service level decrease by approximately 2·5% or more when demand seasonality increases from 2·2 to 3 (Fig 8B).

## Discussion

### Findings

This study reports clear evidence that substantial stock-outs of life-saving health products occurred in Zambia’s public health facilities in the first quarter of 2010. This is noteworthy because these products were available in the central warehouse at that time, and strict adherence to a max-min inventory control policy used in many low-income countries was enforced. These stock-outs can therefore be attributed to that inventory policy, as opposed to procurement or training issues.

Our simple simulation model of this inventory policy offers relatively good predictive accuracy and closely reproduces key features of actual field stock-outs (Fig 5). Sensitivity analyses reveal key drivers of these stock-outs: First, this policy ignores demand seasonality, even though the need for malaria medicines and other health products may be driven by seasonal events (e.g., flooding, changes in water quality, harvest-related exposures, etc…). Specifically, its replenishment targets fail to anticipate upcoming predictable changes in demand (Fig 6). Secondly, these targets also ignore predictable changes in delivery lead-times over time (due to seasonal flooding) and across health facilities (due to local variations in transportation resource availability). Finally, they rely on past consumption as opposed to demand data for predicting future demand. This may create a negative self-perpetrating cycle whereby historical stock-outs are ignored, resulting in insufficient replenishment quantities and increased likelihood that more stock-outs will subsequently occur. In addition, these flaws also result in excessive inventory costs. Specifically, seasonal demand increases are accounted for reactively and seasonal lead-time increases are ignored, so that inventory covering multiple months of demand occupies limited facility storage space after demand peaks. This is particularly wasteful because stocks can be replenished on a monthly basis (Figs 6 and 7). Because of the predictable cyclical nature of stock-outs and excessive inventory costs that are caused by max-min inventory control for life-saving medicines, this policy arguably constitutes a political liability.

The implementation of these inventory policies may nevertheless have been beneficial in many resource-constrained environments in the past, because they facilitate adherence to explicit and consistent management rules by different supply chain actors with limited time and technical capabilities (Fig 1). They are relatively easy to understand and execute for facility staff, and may thus be argued to promote ownership and transparency. Finally, medicines for chronic conditions such as HIV anti-retrovirals have little demand seasonality, and tend to be distributed in facilities with better delivery access. For these medicines, max-min inventory policies are thus unlikely to generate the substantial and systematic stock-outs highlighted here for anti-malarials. These policies are still likely to cause inefficient and excessive stock levels for all products however, because they ignore differences in access conditions, both over time and across facilities.

Our sensitivity analyses suggest that the potential to improve max-min inventory policies through simple changes in parameter values or calculation methods is limited. While increasing the multiple of average monthly issues determining the replenishment target does improve service level, this also increases yearly average and maximum inventory levels by approximately 1:1 and 2:1 ratios, respectively (Fig 7). Even if this change were feasible at all given storage capacity constraints, it would thus be both costly and inefficient. Among the options recommended by USAID | DELIVER for improving the inventory management of anti-malarials [14], none reduces unsatisfied demand below 5% for average lead-times larger than or equal to the baseline value estimated from field data (Fig 8A). Notably, all recommended methods except one result in markedly lower service levels when demand seasonality increases, even though this is the primary feature they are designed to address. One of these methods (using past month of data only), by increasing the average maximum inventory level to approximately six months of demand, would arguably do more harm than good (Fig 8E and 8F). Considering the next three calendar months of the previous year would increase service level to about 95% over a large range of demand seasonality scenarios (Fig 8A and 8B). This estimate may be optimistic however because simulations results do not reflect potential demand estimation errors, and 5% of unsatisfied demand for a life-saving drug under assumed conditions of infinite central supply still seems cause for concern. That service level could also be substantially reduced by any increasing trend in demand, due to its reliance on historical demand data almost one year old.

### Study strengths and limitations

The age of our dataset raises the question of whether the assumptions and conclusions of this paper are still relevant. A first positive confirmation is that as of writing the exact same max-min inventory replenishment policies considered in this paper are being used in Zambia’s public health facilities, which was established by both field observations and an official statement recently issued by the Managing Director of Zambia’s Medical Stores [19]. In addition, the specific inventory management policies considered here are currently being used or recommended for use much beyond Zambia [12,14]. Confirmations that strong seasonality patterns still characterize the demand for malaria products and that public pharmaceutical distribution still involves long and variable lead-times in that country at present are also straightforward [19], and these observations also seem to widely apply beyond Zambia. Finally, a key benefit of our methodology involving both model validation and sensitivity analysis is precisely the ability to establish the robustness of our findings in a wide variety of conditions beyond those observed in 2009/2010 in Zambia. Indeed, our sensitivity analysis results shown in Figs 7 and 8 provide insights applicable to drastically different demand environments, supply lead-time conditions and inventory replenishment policies than are reflected by our original dataset. These observations all suggest that the key findings reported in this study remain applicable in Zambia and the many other countries still using max-min inventory policies to manage the distribution of their health products today.

Our field evaluation of stock-outs relies on stock control cards (Fig 1A) documenting inventory located in the pharmacy but not in the clinical area of facilities. We believe resulting discrepancies to be inconsequential because official facility management guidelines mandate that health products be dispensed out of facility pharmacies in small quantities and only as needed. Notably, current inventory control policies do not take clinical area stock into account (Fig 1B).

Finally, malaria products present a combination of challenges (demand seasonality, distribution to remote facilities) that seem representative of many other essential medicines. Our conclusions may not apply to all other health products however, in particular products with cold chain requirements and very short shelf lives may deserve specific investigations.

### Implications for supply chain practice and research

This study points to a contradiction of all max-min inventory management methods considered here, which are currently used for many health products throughout SSA: under a “pull” model [20] whereby facility/clinic staff compute replenishment quantities themselves, it is debatable how much ownership is developed by merely executing imposed basic calculations. Perceived system transparency due to simple and easy to understand inventory calculations is also unclear, because order quantities can be subsequently rationed down due to central stock-outs as part of a process that is not transparent. Finally, because these methods rigidly restrict the input of health facilities to historical data, they do not even achieve the key potential benefit of a decentralized distribution system, namely the acquisition of rich local information about future supply and demand conditions. Because health facilities are often under-staffed [10], the cost of local staff involvement in inventory calculations may thus outweigh its benefits. However, in a “push” model [20] whereby health facilities merely provide input data and replenishment quantities are centrally determined (or the so-called “informed push” variant whereby a private or internal operator focused on logistics regularly visits facilities to count stock and issue replenishment quantities), potential constraints on technical capabilities of facility staff become less relevant to inventory control. For such a model, the shipment calculation and forecasting methods studied here thus seem overly simplistic and their inventory/service level performance unacceptable.

Our simulation study demonstrates that a root cause for the concerning stock-outs and inefficient inventory levels observed is the underlying methodology used by the current max-min policies considered for forecasting future demand. Indeed, this methodology is ad-hoc and fails to properly capture the seasonality, trend and variations across facilities of both demand and delivery lead-times. These problems have led other researchers to propose alternative forecasting methodologies based on seasonality indices that are specific to demand for malaria commodities [15]. Given the higher conceptual complexity and computational requirements of these methods however, it seems doubtful that they can be widely implemented in peripheral facilities to improve inventory replenishment without substantial investments in computer hardware, software and training. These investments may be disease-specific, and would need to be complemented with methods and processes to also forecast delivery lead-times. Fortunately however, several robust and scalable generic forecasting software implementing time-series analysis methods are now available and widely used in private sector supply chains because they enable distribution organizations to successfully address these exact forecasting challenges across a wide range of different products [16]. While these solutions are relatively user friendly, they do require a minimum level of appropriate quantitative training and a reliable computer system, so that in the short term their systematic use only seems practical at the central warehouse level, or by a private third party logistics provider. As a result, a key opportunity highlighted by our study is to implement tested industrial solutions and develop associated basic capabilities for the forecasting of local demand and local delivery lead-times with seasonality and trend at the central or perhaps regional level, and to modify current inventory replenishment practices in order to leverage this forecasting capability.

Such an initiative would be synergistic with, but not dependent upon, the recent unprecedented increase in availability and affordability of wireless communications and portable computing devices in SSA, which creates an historic, ground-breaking opportunity to improve pharmaceutical supply chains and reduce stock-outs of medicines. Specifically, this development conceptually enables the cost-effective digital recording and communication of all inventory-related transactions using ergonomic portable devices (e.g., smartphones with barcode scanner, tablets). This supply-chain digitization, pervasive in higher-income settings [21], would (i) relieve overburdened health facility staff from time-consuming and inefficient paperwork and stock counting activities, thus improving their productivity; (ii) create seamless visibility of local inventory levels, demand patterns and delivery lead-times, thus transforming critical yet currently problematic supply chain activities including performance management and procurement needs calculations; and (iii) facilitate the implementation of the forecasting and inventory control methods described above, thus achieving higher service levels and lower inventory costs.

While recent positive initiatives involving weekly stock reporting for anti-malarials using SMS technology should be noted [9,22], these arguably fall short of the possible radical transformation just described. This and specific results in this paper prompt an examination of the processes by which innovations and technical recommendations for global health supply chains are currently generated: in stark contrast with drug prescriptions and medical procedures for individual patients, widespread guidelines for distributing inventory of health products affecting entire patient populations are currently designed and disseminated by publicly-funded private firms without conflict of interest statements, without much reliance on independent peer-reviewed research, and under insufficient technical oversight. We hope that this work may help convey the need for independent peer-reviewed research on pharmaceutical supply chains in low-income countries.

## Supporting Information

S1 File Supplementary Material for the Paper “The Impact of Inventory Management on Stock-outs of Essential Drugs in Sub-Saharan Africa: Secondary Analysis of a Field Experiment in Zambia”. (PDF)
