# Supplementary material for: The Impact of Inventory Management on Stock-Outs of Essential Drugs in Sub-Saharan Africa: Secondary Analysis of a Field Experiment in Zambia
Source: PLoS One. 2016 May 26;11(5):e0156026. doi: 10.1371/journal.pone.0156026 (PMC4882057; doi:10.1371/journal.pone.0156026)
Supplement: S1 File — (PDF) [file pone.0156026.s001.pdf]

Supplementary Material for the Paper "The Impact of Inventory Management on Stock-outs of Essential Drugs in Sub-Saharan Africa: Secondary Analysis of a Field Experiment in Zambia"

(Document Reference: S1 File)

May 15, 2016

Ngai-Hang Z. Leung[1], Ana Chen[2], Prashant Yadav[3], Jérémie Gallien[4*]

1 Tepper School of Business, Carnegie Mellon University, Pittsburgh, PA, USA. Current affiliation: Department of Management Sciences, College of Business, City University of Hong Kong, Hong Kong, China, **2** Analytics Operations Engineering, Boston, MA, USA, **3** William Davidson Institute, Ross School of Business and School of Public Health, University of Michigan, Ann Arbor, MI, USA, **4** London Business School, Regent's Park, London NW1 4SA, United Kingdom

* jgallien@london.edu

A. Results from the 2009/10 supply chain pilot

The main impact evaluation methodology used for the 2009/2010 supply chain pilot involved field measurements of the stockouts experienced during the fourth quarter of 2009 by clinics in the two treatment arms (A and B districts) and the control arm (control districts) of the experiment. Specifically, a team of field data analysts visited all these clinics at some point during the first and second quarters of 2010 in order to determine from their stock control cards the number of days in Q4 2009 during which each of the four drug formats AL6, AL12, AL18 and AL24 was unavailable in each clinic. The main statistics and results of the statistical tests evaluating the differences in these number of days of stockouts for these four products and the three arms of this experiment are shown in Table a.

Table a: Average number of days of stock-out for artemether-lumefantrine (AL) products during Q4 2009 in health clinics of the control, intervention A and intervention B districts of the 2009/10 pilot (from Vledder et al.).[9]
P-values of difference adjusted for design effect at district level as well as controls for stratification variables.

Product	Control districts (days)	A districts (days)	P-value for difference from control districts	B districts (days)	P-value for difference from control districts
AL 6	29·2 (n=69)	17·6 (n=70)	0·238	4·6 (n=63)	0·002
AL 12	16·6 (n=69)	11·6 (n=70)	0·484	0·2 (n=63)	0·009
AL 18	19·7 (n=69)	15·7 (n=70)	0·537	2·0 (n=63)	0·03
AL 24	24·3 (n=69)	14·1 (n=70)	0·192	1·1 (n=63)	0·005

B. Additional model validation

The collection of stock card data that forms the basis of the field measurement of stockouts and model validation results reported in the paper was distinct and independent from the data collection which was organized as part of the impact evaluation activities of the 2009/2010 supply chain pilot, which is described in Section A and summarized in Table a above. Specifically, the respective sets of clinics for which data was obtained were distinct, and the data collection teams involved different individuals employed by separate organizations. More importantly, the data collected to evaluate the impact of the 2009/10 pilot consisted of the number of days with stockout during Q4 2009, whereas the data collected as part of the research reported in the paper consisted of all the inventory transactions documented on stock control cards. The latter is a more detailed dataset which enabled for example the estimation of how the proportion of clinics stocking outs changed over the course of several quarters, as shown in Fig 5. Because the number of stockout days during Q4 2009 can also be predicted by our simulation model however, the 2009/10 pilot evaluation data summarized in Table a offers an opportunity for another model validation exercise that is independent from the one reported in Fig 5. To that end, Table b compares the number of days without stock during Q4 2009 simulated with the model described in the paper with the corresponding actual values estimated from the field measurements organized for the evaluation of the 2009/10 pilot (also seen in Table a). Specifically, the statistical experiment reported in Table b considers the distribution of the sample average of stockout days in Q4 2009 over 63 simulated replications of policy 4 x I [-3,0] (the same sample size used for the evaluation of the clinics in the B districts reported in Table a), and evaluates the percentile of the actual sample average from the field with respect to that simulated distribution. In other words, it evaluates the likelihood of observing the field data collected under the assumption that the field data was drawn from the simulated distribution.

Table b: Measured and simulated statistics for the number of days of stock-out for artemether-lumefantrine (AL) products during Q4 2009.
Actual measurements from surveyed health facilities of intervention B districts of the 2009/10 pilot as reported by Vledder et al.[9] Simulation results obtained from 1000 independent replications of policy 4 x I [-3,0] and 1000000 independent sets of 63 replications for each product.

Product	Average of actual measurements from primary analysis (days)[9]	Simulated 5th percentile (days)	Simulated Median (days)	Simulated 95th percentile (days)	Simulated percentile of actual measurement
AL 6	4·6 (n=63)	0·80	2·32	4·58	95·6
AL 12	0·2 (n=63)	0·00	0·49	1·45	25·8
AL 18	2·0 (n=63)	0·08	0·76	2·01	95·1
AL 24	1·1 (n=63)	0·56	1·98	4·00	19·2

As seen in Table b, actual number of days without stock for AL 12 and 24 are very likely in terms of the corresponding simulated distribution (actual values at the 25·8th and 19·2th simulated percentiles, respectively), and although the simulation model may be underestimating stock-outs for

AL 6 and AL 18 (actual values at the 95·6th and 95·1th simulated percentiles, respectively), the discrepancies of mean values remain small in comparison with the actual and simulated stock-out levels observed for other quarters in Fig 5. These results therefore lend support to the robustness of the validation findings reported in the paper. Namely, the aggregated validation evidence from the paper and this supplementary material suggests that the simulation model predictions are accurate for some products and may be under-estimating stockouts for other products (AL 6 in particular), however the extent of this likely under-estimation bias does not alter the key conclusions of the study.

C. Alternative evaluation of model validity

The main validation test of predictive accuracy reported in the paper considers the actual fraction of clinics stocking out as a random realization by nature/the field, to be compared with the full probability distribution of the same proportion that is generated by the simulation model (Fig 5). We discuss here another legitimate statistical model validation test, namely whether the simulated mean of clinics stocking out can plausibly coincide with the population mean estimated from the actual sample proportion of clinics stocking out. To that end, Fig a displays the same actual and simulated mean proportion of clinics stocking out featured in Fig 5, but also includes a 90% confidence interval on the population mean estimated from the observed actual data.

Figure a: Actual and simulated mean stock-out probabilities for artemether-lumefantrine (AL) products, with 90% confidence interval on the population mean estimated from actual sample proportion of clinics with stockout.

Actual stock-out probabilities estimated from stock control card data as the fraction of surveyed health facilities without stock. Mean simulated stock-out probabilities estimated as the fraction of 100,000 replications without stock. 90% confidence interval constructed as the Wilson score interval for binomial proportion using the sample size shown in Fig 4.

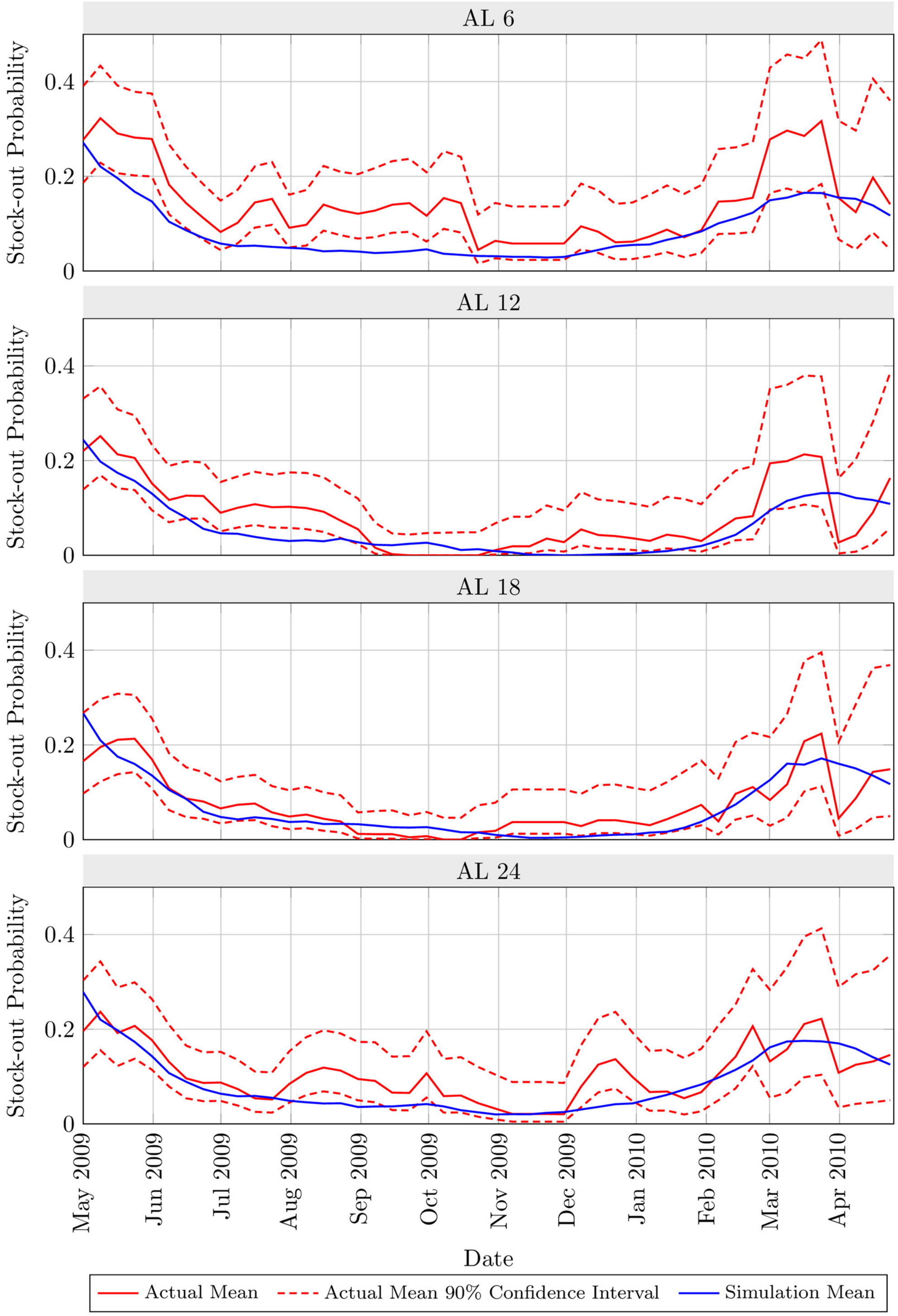

We note that these results lend support to the robustness of the validation results reported in the paper. Specifically, for all drug formats the simulated mean is found to lie most of the time within the 90% confidence interval for the population mean constructed from actual data. Furthermore, these results also suggest that the simulation model may be under-estimating the actual proportion of clinics stocking out of AL6 between May and October 2009.

D. Demand distribution fitting and selection

We describe in this section the data analysis and fitting work that led to the selection of the lognormal distribution with a coefficient of variation of 50% to represent weekly demand in our simulation model. The key steps of this analysis are as follows:

Step 1: Fitting dataset selection

For demand fitting purposes we selected a subset of the stock card transaction dataset described in the Data collection subsection in the Methods section of the paper. Specifically, we selected the 6 health centers in that dataset with the longest period of uninterrupted data. In addition, in each health center we removed the dosage forms for AL products for which some stockout was recorded at some point during the recorded time period. The resulting dataset scope is stated in Table c:

Table c: Demand fitting dataset description.

Health Center	Included Products	Start Date	End Date	Number of Days
Chiwoma	AL 6, 12, 24	06-24-2009	04-04-2010	284
Tembwe	AL 6, 12, 24	05-29-2009	31-01-2010	247
Mapamba	AL 6, 12, 18, 24	06-18-2009	27-02-2010	254
Shem	AL 12, 18, 24	08-04-2009	01-03-2010	327
Chingi	AL 6, 24	05-03-2009	15-01-2010	316
Chozi	AL 6, 12, 18, 24	30-05-2009	28-02-2010	274

Step 2: Mean daily demand estimation

We next estimated the mean daily demand for each day and (health center, product) combination included in the fitting dataset described in Step 1. This was performed in order to subsequently constrain the distribution fitting procedure to only consider daily demand distributions with the same first moment as that inferred from data. This estimation step followed the procedure described in the Data collection subsection of the Methods section of the paper:

- The data for each health center and product is parsed into events $(u^n_{pi}, v^n_{pi}, q^n_{pi})_n$ corresponding to the issue on day u^n_{pi} from the pharmacy to the clinic area in health center i of one or several boxes of product p (AL 6, 12, 18 or 24) containing q^n_{pi} doses (typically 30 or 60, which is the contents of one or two boxes), which covered demand until day v^n_{pi} when the next box of the same product was issued. In order to exclude periods with stockouts, only issues performed when the remaining stock is larger than one box are considered;
- Whenever possible, raw consumption R_{tpi} is calculated for each day t, health center i and product p as

$$R_{tpi} = q^n_{pi} / (v^n_{pi} - u^n_{pi}) \text{ for each day t in interval } [u^n_{pi}, v^n_{pi}];$$

- To smooth discontinuities and estimate censored demand, a triple centered moving average operator with successive half-widths 40, 30 and 20 days of non-censored data is applied to the time series $(R_{tpi})_t$ associated with each health center i and product p in the dataset. This transformation generates smoothed and uncensored time series $(C_{tpi})_t$ of consumption data;
- Because of reported common demand substitution between AL products (which contain identical pills), their individual daily consumption estimates $(C_{tpi})_t$ are converted into adult doses then summed across products, and that sum is then split again between the four different products using proportions equal to the fractions α_p of issues observed in the entire stock card dataset (12% for AL 6, 13.5% for AL 12, 22.5% for AL 18, 52% for AL 24). This resulted in an estimate of mean daily demand D_{tpi} for each day t, product p and health center i in the fitting dataset:

$$D_{tpi} = \alpha_p \times \sum_{p\in\{6,12,18,24\}} (p/24) \times C_{tpi}.$$

Step 3: Maximum likelihood fitting

We finally performed maximum likelihood estimation in order to assess the fit of various parametric families of probability distributions to represent daily demand for the fitting dataset constructed in Step 1. We specifically considered the following families of probability distributions with one or two parameters: Poisson; geometric; negative binomial; normal and lognormal. For the Poisson and geometric families of distribution, we evaluated for each health center and product combination in the fitting dataset the log-likelihood of observing the recorded data if the demand for each day were to follow the corresponding distribution with a mean constrained to be equal to D_{tpi}. Considering the sequence of data events $(u^n_{pi}, v^n_{pi}, q^n_{pi})_n$ defined in Step 2 above, we specifically evaluated

$$\sum_n \log P\left(\sum_{t=u_{pi}^n}^{v_{pi}^n} X_t(D_{tpi}) = q_{pi}^n\right),$$

where P denotes probability and $X_t(D_{tpi})$ is a random variable with mean D_{tpi} following the distribution considered (Poisson or geometric). For the negative binomial family of distribution, we solved through line search the optimization problem

$$\max_\beta \sum_n \log P\left(\sum_{t=u_{pi}^n}^{v_{pi}^n} X_t(D_{tpi},\beta) = q_{pi}^n\right),$$

where $X_t(D_{tpi},\beta)$ is a random variable following a negative binomial distribution with mean D_{tpi} and parameter β. Specifically, we considered two parameterizations for this family, setting β to be either the success probability or the coefficient of variation (COV) of the negative binomial distribution. For the lognormal and normal families of distribution, we solved through line search the optimization problem

$$\max_\beta \sum_n \log P\left(q_{pi}^n - 0.5 \le \sum_{t=u_{pi}^n}^{v_{pi}^n} X_t\big(D_{tpi},\beta\big) \le q_{pi}^n + 0.5\right),$$

where $X_t(D_{tpi},\beta)$ is a random variable following the distribution considered with mean D_{tpi} and parameter β. In the case of the normal family of distributions, we set β to be either the standard

deviation or the coefficient of variation (COV) of the distribution. In the case of the lognormal family of distributions, we set β to be the coefficient of variation (COV).

For all the log-likelihood estimation or optimization procedures described above, we assumed the random variables corresponding to demand in different days to be independent. The results of the likelihood calculation or optimization procedures just described are summarized in Table d.

Table d: Log-likelihood calculation and optimization-based estimation results.

		Geometric	Poisson	Negative Binomial Fixed COV		Negative Binomial Fixed p		Normal Fixed COV		Normal Fixed Sigma		Lognormal Fixed COV	
Health Center	AL Product	Log-likelihood	Log-likelihood	Log-likelihood	Parameter	Log-likelihood	Parameter	Log-likelihood	Parameter	Log-likelihood	Parameter	Log-likelihood	Parameter
Chiwoma	6	-54.91	-73.08	-52.23	1.90	-52.50	0.18	-51.96	2.00	-56.41	5.00	-52.93	2.40
Chiwoma	12	-15.41	-17.90	-14.55	2.20	-15.07	0.30	-14.59	2.40	-16.17	3.60	-14.74	2.40
Chiwoma	24	-50.53	-62.52	-49.86	1.50	-50.14	0.25	-50.16	1.70	-53.49	5.00	-49.96	1.70
Tembwe	6	-125.57	-267.57	-89.39	3.10	-90.30	0.04	-87.99	4.00	-89.42	30.00	-93.89	9.80
Tembwe	12	-93.57	-294.99	-79.33	2.40	-79.53	0.06	-78.55	4.40	-137.75	30.00	-81.80	3.40
Tembwe	24	-76.99	-113.81	-75.16	1.60	-74.92	0.15	-73.94	1.80	-76.68	27.30	-76.80	2.00
Mapamba	24	-235.17	-584.12	-178.65	2.70	-173.03	0.06	-184.17	5.80	-197.57	30.00	-181.71	4.10
Mapamba	18	-119.99	-261.57	-92.27	2.80	-92.20	0.09	-89.95	5.80	-97.78	30.00	-96.94	3.20
Mapamba	12	-220.14	-531.84	-165.55	2.80	-162.84	0.06	-153.51	5.10	-172.03	30.00	-174.43	4.20
Mapamba	6	-356.08	-988.15	-249.50	3.00	-243.01	0.05	-248.58	7.30	-297.57	30.00	-258.52	5.30
Shem	12	-10.85	-11.46	-10.55	2.50	-10.61	0.38	-10.82	2.20	-10.87	0.90	-10.14	3.40
Shem	18	-11.52	-10.78	-12.53	2.30	-10.80	0.99	-9.64	0.70	-8.35	0.10	-9.74	0.70
Shem	24	-15.90	-17.08	-15.14	2.80	-14.94	0.27	-14.93	2.30	-14.06	0.90	-15.17	5.10
Chingi	6	-17.71	-19.41	-17.55	1.70	-18.06	0.44	-17.46	1.50	-19.66	3.60	-17.38	1.50
Chingi	24	-27.09	-30.12	-27.15	1.90	-26.40	0.28	-26.88	1.70	-27.01	3.90	-27.17	2.90
Chozi	24	-116.99	-193.36	-112.82	1.60	-113.37	0.13	-119.30	2.20	-119.97	30.00	-112.43	2.00
Chozi	18	-21.37	-29.08	-16.84	3.20	-17.19	0.14	-16.52	4.20	-17.47	9.30	-17.32	3.60
Chozi	12	-52.46	-91.25	-41.40	2.80	-42.30	0.06	-39.75	2.30	-41.98	30.00	-42.55	6.80
Chozi	6	-74.89	-94.90	-74.46	1.40	-74.62	0.22	-74.99	1.50	-75.87	12.70	-75.01	1.60
Mean		-89.32	-194.37	-72.37	2.33	-71.68	0.22	-71.77	3.10	-80.53	16.44	-74.14	3.48

On the basis of the results shown in Table d, we discarded the geometric and Poisson families of distributions because of the low value of their mean log-likelihood. The differences in average log-likelihood values between the other fitted families of distributions were not considered significant however, so that the lognormal distribution with fixed COV was selected among them because of its appealing analytical properties. Out of concern that the average estimated value for the COV parameter of the lognormal and normal families of distribution were driven by outliers and/or discrete approximations used with continuous distributions, we selected the average coefficient of variation of daily demand estimated for the negative binomial distribution, that is 2.33 or 233%. This corresponds to a COV for weekly demand approximately equal to 0.5 or 50%. The model output validation results reported in the paper and Sections B and C of this document suggest that these choices were appropriate.
